# Minimization and optimization of α-amylase terminator for heterologous protein production in *Bacillus licheniformis*

**DOI:** 10.1186/s40643-022-00597-1

**Published:** 2022-10-10

**Authors:** Yi Rao, Jingyao Yang, Jiaqi Wang, Xinyuan Yang, Mengxi Zhang, Yangyang Zhan, Xin Ma, Dongbo Cai, Zhangqian Wang, Shouwen Chen

**Affiliations:** 1 State Key Laboratory of Biocatalysis and Enzyme EngineeringEnvironmental Microbial Technology Center of Hubei ProvinceCollege of Life Sciences, Hubei University, 368 Youyi Avenue, Wuchang District, Wuhan, 430062 Hubei People’s Republic of China; 2 Hubei Engineering Research Center for Deep Processing of Green Se-Rich Agricultural Products, School of Modern Industry for Selenium Science and Engineering, National R&D Center for Se-Rich Agricultural Products Processing, Wuhan Polytechnic University, Wuhan, 430023 People’s Republic of China; 3 Fujian Provincial Key Laboratory of Eco-Industrial Green Technology, College of Ecological and Resource Engineering, Wuyi University, Nanping, 354300 Wuyishan People’s Republic of China

**Keywords:** Intrinsic terminator, Synthetic biology, Protein expression, *Bacillus licheniformis*

## Abstract

Terminators serve as the regulatory role in gene transcription termination; however, few researches about terminator optimization have been conducted, which leads to the lack of available and universal terminator for gene expression regulation in *Bacillus*. To solve this problem and expand synthetic biology toolbox of *Bacillus licheniformis*, the terminator T1 of endogenous α-amylase gene (*amyL*) was characterized in this research, with a termination efficiency of 87.81%. Then, we explored and optimized the termination strength of terminator T1 from four aspects: the distance between stop codon and terminator, GC content at the bottom of stem structure, loop size, and U-tract length, and the best terminator T24 was attained by combination optimization strategy, which termination efficiency was increased to 97.97%, better than the commonly used terminator T7 (T7^P^) from *Escherichia coli*. Finally, terminator T24 was applied to protein expression, which, respectively, led to 33.00%, 25.93%, and 11.78% increases of green fluorescence intensity, red fluorescence intensity, and keratinase activity, indicating its universality in protein expression. Taken together, this research not only expands a plug-and-play synthetic biology toolbox in *B. licheniformis* but also provides a reference for the artificial design of versatile intrinsic terminator.

**Graphical Abstract:**

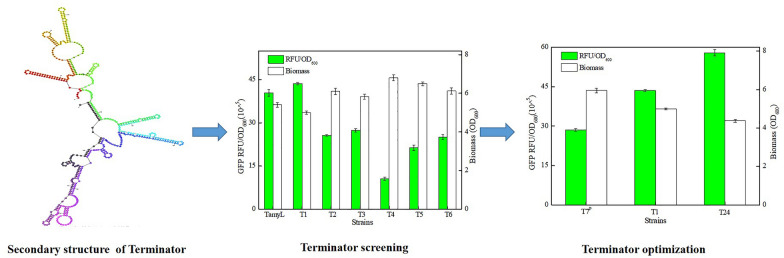

**Supplementary Information:**

The online version contains supplementary material available at 10.1186/s40643-022-00597-1.

## Introduction

With the flourishing development of synthetic biology, microbial genetic systems have gradually made great strides in the direction of programming and simplification, which enables biological systems to refine metabolic pathways of target products and achieve the controllability of gene expression (Gibson et al. 2010; Zong et al. 2017). The realization of this process mainly relies on the leapfrog development of gene editing technology (CRISPR/Cas system) and establishment of a series of standardized and normalization plug-and-play synthetic biology toolboxes (logic gates, oscillators, etc.) (Knott 2018; Kang et al. 2021; Lin et al. 2016). The development of these toolkits mostly relies on the transcriptional and post-transcriptional regulatory elements, including promoters and ribosome binding sites (RBS) (Jervis et al. 2019; Sauer et al. 2018). In addition, researchers recently developed several types of gene regulation tools by using transcription termination regulatory element (terminator) to expand synthetic biology toolbox, and their results implied that terminator could also be served as a candidate element for the construction of standardized plug-and-play toolkit in biological system (Curran et al. 2013; Pfleger et al. 2006).

Terminator is a DNA sequence located downstream of coding gene, which has the function of gene transcription termination and mRNA stabilization, and it was generally divided into 2 categories: Rho-dependent and Rho-independent terminators (intrinsic terminator) (Hironori Otakaa et al., 2011; Jain et al. 2019). In prokaryotes, the majority of terminators are intrinsic terminators, and previous researches were mainly focused on the prediction and identification of intrinsic terminators. For instance, David et al., predicted the terminators of 2,200 annotated genes, and 1075 of which were intrinsic terminators in *E. coli* K12 (Elena et. al., 2001). Then, Robert et al., found that intrinsic terminator has a specific structure feature, which is mainly composed of a GC-rich stem-loop structure and a 6–8 nt U-tract (Peters et al. 2011). On this basis, people have screened different intrinsic terminators and evaluated the effects of GC content at the bottom of stem-loop structure, Gibbs free energy ΔG, and U-tract length on their performances (He et al. 2020; Cui et al. 2021; Chen et al. 2013) (Additional file 1: Table S3). Among them, He et al., have analyzed 12 terminator sequences and implied that GC content at the bottom of stem structure was positively correlated with terminator intensity in *E. coli* (He et al. 2020). In addition, Chen et al., have constructed a terminator library in *E. coli*, established a mathematical model for the prediction of termination efficiency, and found that the Gibbs free energy △G of terminator is moderately or weakly related to its termination efficiency (Chen et al. 2013). Based on the previous results, these studies provided a theoretical basis for selecting suitable terminator to achieve precise regulation of gene expression and metabolic pathway optimization (Curran et al. 2013). However, terminator screening is often cumbersome, and termination strength of natural terminator is quite varied in different genetic backgrounds, due to the terminator specificity. Therefore, terminator modification strategy is regarded as a better way for constructing the universal terminator. At present, terminator has been developed as a plug-and-play toolbox through terminator modification, which played an important role in different expression systems, such as *E. coli*, yeast, and *Pseudomonas putida* (Amarelle et al. 2019; Rossmanith et al. 2018; Uwimana et al., 2017).

*Bacillus licheniformis* DW2 is a protein expression platform bacteria, which has the characteristics of high protein secretion and biological safety (Xiao et al. 2020). However, there are few reports on *Bacillus* terminator screening and optimization in these years. Based on the analysis and statistics of sequence characteristics of 80 endogenous intrinsic terminators in *B. subtilis* 168, Cui et al., have designed 9 terminators, which termination efficiency was greater than 70% in different hosts (Cui et al. 2021). Here, in order to construct a plug-and-play toolkit in *B. licheniformis*, the terminator of endogenous α-amylase gene (*amyL*) from *B. licheniformis* WX-02 was optimized and tested, based on the analysis of impact factors of intrinsic terminator. Taken together, this work provides not only a new plug-and-play regulatory element for *Bacillus* but also a reference for artificially designing excellent terminator.

## Methods and materials

### Strains, plasmids, and cultivation conditions

Strains and plasmids used in this research are listed in Additional file 1: Table S1. *E. coli* DH5α was used for vector construction; *B. licheniformis* DW2 was used as the protein expression host. The plasmid pHY300PLK was applied to construct gene expression vectors with different terminators, and when using episomal expression plasmid pHY300PLK, we first ensured that all sequences were consistent without expression cassettes. *E. coli* and *B. licheniformis* were grown on Luria–Bertani (LB) agar plates or in LB broth at 37 °C, 230 rpm, with 20 mg/L tetracycline addition when necessary. For GFP and RFP expression, strains were cultivated in 250 mL flask with 50 mL LB for 24 h. For keratinase expression assay, strains were cultivated in keratinase production medium (2% glucose, 1.5% yeast extract, 1% tryptone, 1% NaCl, 0.6% (NH_4_)_2_SO_4_, 1% corn steep liquor, 0.3% K_2_HPO_4,_ and 1% soybean meal, pH 7.2) for 48 h. All the fermentation experiments were repeated at least three times.

### Construction of gene expression vectors with different terminators

The *gfp* expression plasmids with different terminators were constructed basing on vector pHY/pP_ylB_-GFP, according to our previously reported method (Xiao et al. 2020), and construction procedure of pP_ylB_-GFP-T1 (pHY/YG-T1) mediating by terminator T1 was served as an example. In brief, the expression vector harboring terminator T1 was amplified from vector pP_ylB_-GFP-TamyL by corresponding primers (Additional file 1: Table S2), and self-connected using ClonExpress One Step Cloning Kit (Vazyme Biotech Co., Ltd, Nanjing, China), following to the manufacturer’s instructions, resulting in plasmid pP_ylB_-GFP-T1. In addition, the construction map and sequence of vector pHY/PylB-GFP-Tn are provided in Additional file 1: Figure S2 and Table S4, respectively. Similarly, a series of GFP, RFP, and keratinase expression vectors were constructed with the same method.

### Determination of termination efficiency

Termination efficiency was measured according to the previously reported method (Cui et al. 2021), and the termination efficiency of terminator T1 was measured as an example. DW2/YG-RFP was acted as the reference strain, as no terminator existed between genes *gfp* and *rfp*. Terminator T1 was added between genes *gfp* and *rfp*, resulting in the test strain DW2/YG-T1-RFP. Then, green and red fluorescence intensities of DW2/YG-T1-RFP and DW2/YG-RFP were measured, and the corresponding termination efficiency of T1 was calculated by the following equation:

$${\text{TE(\% )}} = \left\{ {{1} - \left. {\frac{{{\text{RFP}}^{{{\text{tes}}}} {\text{/RFP}}^{{{\text{ref}}}} }}{{{\text{GFP}}^{{{\text{tes}}}} {\text{/GFP}}^{{{\text{ref}}}} }}} \right\}} \right..$$

### Analytic methods

Cell biomass was determined by determining OD_600_ or dry cell weight of cell broth. The green and red fluorescence intensities were measured by Multi-Mode Microplate Reader (SpectraMax iD3, Molecular Devices), according to our previously reported method (Xiao et al. 2020). Keratinase activity assay was conducted according to the previously reported method, with soluble keratin as substrate (Peng et al. 2021). Gene transcriptional levels were analyzed by RT-qRCR, and *16S rDNA* was served as the reference gene (Cai et al. 2020).

### Statistical analyses

Data are presented as the mean ± standard deviation for each sample point. All data were conducted to analyze the variance at *p* < 0.05 and *p* < 0.01, and the mean values were compared by applying a *t* test, using the software package Statistica 6.0.

## Results and discussions

### Identify and characterize the minimal intrinsic terminator of gene amyL

Previously, the 501 bp downstream of endogenous α-amylase gene *amyL* from *B. licheniformis* WX-02 was generally used as the regulatory element of gene transcription termination. Here, based on the characteristics of reported intrinsic terminator (Peters et al. 2011), we want to identify and characterize the minimal terminator (TamyL) of *amyL*. The secondary structure of TamyL was predicted by RNA Mfold online website (http://www.unafold.org/RNA_form.php) (Additional file 1: Fig. S1), and six putative α-amylase terminators were attained based on the features of intrinsic terminator (T1-T6), which sequences are provided in Table 1. Then, six GFP expression plasmids with different terminators, pHY/pP_ylB_-GFP-(T1-T6) (YG-(T1-T6)), were constructed and then electro-transformed into *B. licheniformis* DW2 to obtain GFP expression recombinant strains DW2/YG-T1, DW2/YG-T2, DW2/YG-T3, DW2/YG-T4, DW2/YG-T5, and DW2/YG-T6, respectively. Then, all these strains were cultivated in LB medium, as well as control strain DW2/YG-TamyL with original terminator.

**Table 1:** The design sequences of different terminators

Terminators	Sequence
T1	CGGATTTCCTGAAGGAAATCCGTTTTTTTA
T2	TTTGATTACATTTTATAATTAAT
T3	ACAAAGTGTCATCAGCCCTCAGGAAGGACTTGCTGACAGTTTGA
T4	GACGGTATCGCGGGTGATCAATCATCCTGAGACTGTG
T5	ATGAATCTGTTAACGGGAATCAG
T6	CGCGAGCTGGACCGTCATCATTATGCTTTGCAGCTTGTC
T7	CCGGATTTCCTGAAGGAAATCCGTTTTTTTA
T8	CCCCCGGATTTCCTGAAGGAAATCCGTTTTTTTA
T9	CCCCCCCGGATTTCCTGAAGGAAATCCGTTTTTTTA
T10	CCCCCCCCCGGATTTCCTGAAGGAAATCCGTTTTTTTA
T11	CCCCCCCCCCCGGATTTCCTGAAGGAAATCCGTTTTTTTA
T12	CCCCCCCCCCCCCGGATTTCCTGAAGGAAATCCGTTTTTTTA
T13	CCCCCCCCCCCCCCCCCGGATTTCCTGAAGGAAATCCGTTTTTTTA
T14	CCCCCCCCCCCCCCCCCCCCCGGATTTCCTGAAGGAAATCCGTTTTTTTA
T15	CGGCGCATTTCCTGAAGGAAATGCGCCGTTTTTTTA
T16	CATATTTCCTGAAGGAAATATGTTTTTTTA
T17	CGGATTTCCTTGACCGGAAATCCGTTTTTTTA
T18	CGGATTTCCTTTGACCCGGAAATCCGTTTTTTTA
T19	CGGATTTCCTTTTGACCCCGGAAATCCGTTTTTTTA
T20	CGGATTTCCTTTTTGACCCCCGGAAATCCGTTTTTTTA
T21	CGGATTTCCTGAAGGAAATCCGTTTTTCTA
T22	CGGATTTCCTGAAGGAAATCCGTTTCTCTA
T23	CGGATTTCCTGAAGGAAATCCGTCTCTCTA
T24	CCCCCCCCCCCGGCGCATTTCCTTTGACCCGGAAATGCGCCGTTTTTTTA
T7^P^	AACCCCTTGGGGCCTCTAAACGGGTCTTGAGGGGTTTTTTGCT

**Fig. 1:**
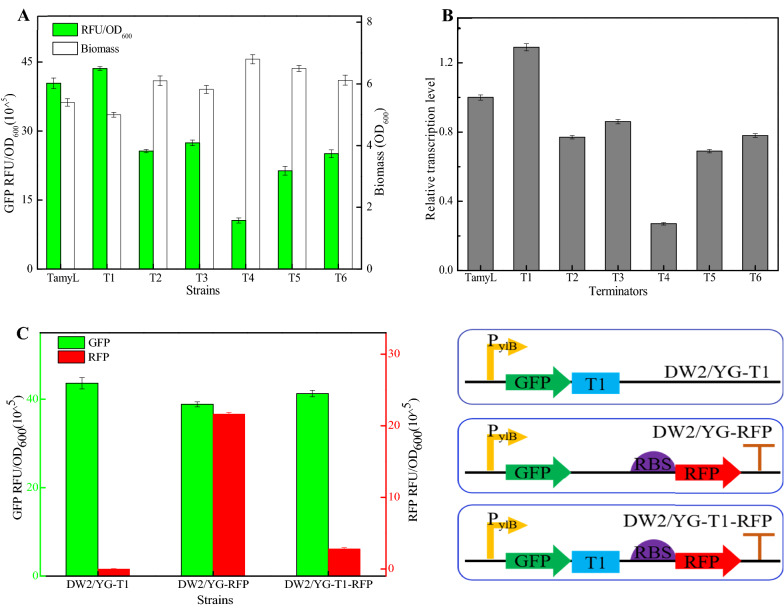
Identification and characterization of α-amylase terminator. **A** Green fluorescence intensities and cell biomasses of GFP expression strains with different terminators (T1–T6). **B** Relative transcriptional levels of *gfp*. **C** Dual-gene reporter system for termination efficiency determination

Based on our results, the cell biomasses were negatively correlated with GFP expression levels among these strains (Fig. 1A), and fluorescence intensities (RFU/OD_600_, GFP) mediated by terminators T1-T6 were 43.58 × 10^^5^, 25.65 × 10^^5^, 27.41 × 10^^5^, 10.55 × 10^^5^, 21.35 × 10^^5^, and 25.05 × 10^^5^, which were 107.92%, 63.52%, 67.88%, 26.13%, 52.87%, and 62.04% of control strain (40.38 × 10^^5^), respectively. Among them, terminator T1 with a size of 30 nt owned the best performance (Fig. 1A). The strong performance of terminator will accelerate the detachment of RNA polymerase, which increased the reuse rate of RNA polymerase, thereby increased the initial transcription amount of target gene, and further resulted in the increase of target gene expression (Ray-Soni et al. 2016); also, the fluorescence intensities were consistent with gene transcriptional levels (Fig. 1B). Meanwhile, the size of terminator T1 was smaller than the lengths of most reported terminators of *Bacillus spp* (Cui et al. 2021) (Additional file 1: Table S5).

Terminators with strong termination efficiency can accelerate the release of RNA polymerase at transcription termination and improve the initial expression level of target gene. Thus, the termination efficiency is one of the important indicators to measure terminator strength (Ray-Soni et al. 2016). Subsequently, DW2/YG-RFP and DW2/YG-T1-RFP were constructed to explored the termination efficiency of terminator T1, using the dual-gene reporter system, in which gene *gfp* and *rfp* are co-expressed, mediated by promoter P_ylB_, and ribosome binding sequence for gene *rfp* is the RBS sequence (5’-GAAACAACAAAGGGGGAGATTTGT-3’) of promoter P_ylB_ (Fig. 1C). Based on our results, the relative green and red fluorescence intensities of DW2/YG-RFP were 38.82 × 10^^5^ and 21.62 × 10^^5^, which of DW2/YG-T1-RFP were 41.24 × 10^^5^ and 2.80 × 10^^5^ respectively (Fig. 1C), and the termination efficiency of T1 was 87.81%, which is greater than those of several natural terminators reported in *Bacillus spp* (Cui et al. 2021) (Additional file 1: Table S5); also, more works should to be done to improve the performance of terminator T1 in future work. In addition, the green fluorescence intensity of DW2/YG-RFP was lower than that of DW2/YG-TamyL, and this might be due to the low release rate of RNA polymerase and efficiency of ribosome utilization in DW2/YG-RFP. This result implied that terminator T1 could prevent the read-through of gene *rfp*. Taken together, our results showed that intrinsic terminator T1 could be used as a candidate element for the development of a plug-and-play synthetic biology toolbox in *B. licheniformis*.

### Effects of distance between stop codon and terminator on the strength of terminator T1

Based on the above results, we want to further improve the performance of terminator T1. Previously, Liu et al., implied that the optimal 3’-UTR was attained when 12 nt cytosines were inserted between stop codon and 3’-UTR (Deng et al. 2019). Here, the effects of distance between stop codon and terminator on terminator T1 performance were analyzed, and 1, 4, 6, 8, 10, 12, 16, 20 cytosines were, respectively, inserted into 5’-end of T1, attaining different terminators T7–T14 (Table 1).

**Fig. 2:**
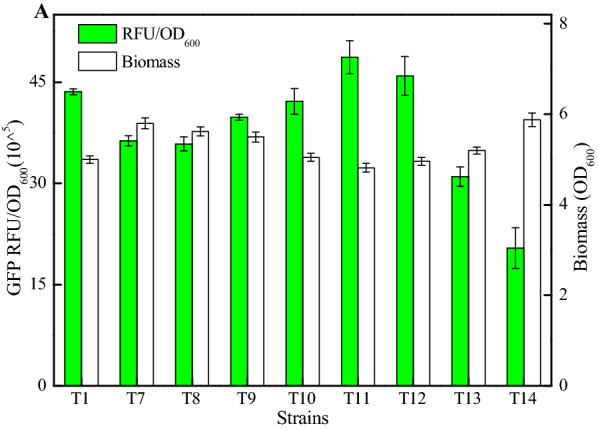
Effect of distance between stop codon and terminator on intrinsic terminator T1. **A** Green fluorescence intensities and cell biomasses of recombinant strains with different terminators (T7–T14)

Subsequently, a series of recombinant strains DW2/YG-(T7-T14) were constructed, and green fluorescence intensities of which strains were determined. Based on our results, the fluorescence intensities (RFU/OD_600_, GFP) mediated by T7-T14 were 36.31 × 10^^5^, 35.84 × 10^^5^, 39.82 × 10^^5^, 42.16 × 10^^5^, 48.70 × 10^^5^, 45.93 × 10^^5^, 31.00 × 10^^5^, and 20.42 × 10^^5^ (Fig. 2A). Compared to terminator T1 (43.58 × 10^^5^), better performances were attained by DW2/YG-T11 and DW2/YG-T12, and the distance between stop codon and terminator T1 were 10 and 12 cytosines, respectively. Meanwhile, the expression efficiency of terminator T1 was significantly reduced when the distance between stop codon and terminator T1 was shorter than 6 nt or longer than 12 nt (Fig. 2A). However, the law of its effect on terminator strength was not consistent with the previous report on 3′-UTR (Deng et al. 2019); we speculated that it may be caused by the different characteristics of terminator and 3'-UTR. Overall, our results indicated that the distance between stop codon and terminator affected terminator performance, and the terminator strength could be fine-tuned by changing the distance between stop codon and terminator.

### The effect of stem-loop structure on the strength of intrinsic terminator T1

**Fig. 3:**
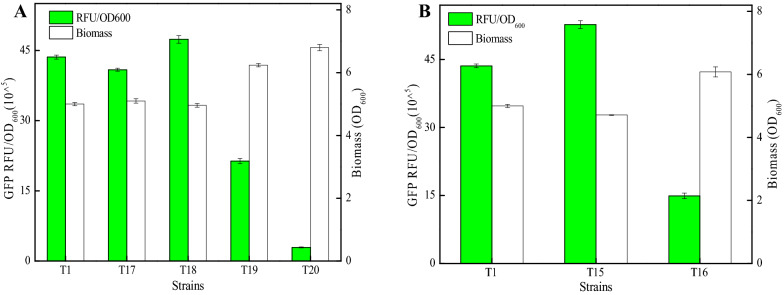
Effect of stem-loop structure on intrinsic terminator T1. **A** Green fluorescence intensities and cell biomasses of strains with different terminators (T15–T16). **B** Green fluorescence intensities and cell biomasses of recombinant strains (T17–T20)

Previously, Yang et al., have reported that GC content of stem-loop structure affects termination efficiency of terminator, and GC content at the bottom of stem-loop structure was positively correlated with terminator strength (Yang et al. 2017). Then, the effect of stem-loop structure on the performance of intrinsic terminator T1 was explored, and terminators with increased and decreased GC contents at the bottom of stem-loop structure were designed (T15 and T16), based on terminator T1 (Table 1). Subsequently, recombinant strains DW2/YG-T15 and DW2/YG-T16 were constructed, and the fluorescence intensity (RFU/OD_600_, GFP) of DW2/YG-T15 was 52.68 × 10^^5^ (Fig. 3A), increased by 20.88% compared to DW2/YG-T1. However, 65.79% decrease of green fluorescence intensity was attained by DW2/YG-T16 (14.91 × 10^^5^). Therefore, our results indicated that GC content at the bottom of stem-loop structure was positively correlated with terminator strength, which was consistent with the previously reported research (Yang et al. 2017).

Currently, the effect of loop size of stem-loop structure on terminator performance has not been analyzed. Here, based on terminator T1, terminators with loop sizes from 8 to 14 nt were designed, named as T17–T20 (Fig. 3B, Table 1). Subsequently, the corresponding recombinant strains DW2/YG-(T17–T20) were constructed, and fluorescence intensities (RFU/OD_600_, GFP) mediated by terminators T17–T20 were 40.87 × 10^^5^, 47.37 × 10^^5^, 21.37 × 10^^5^, and 2.91 × 10^^5^, respectively. It was found that only loop size with 10 nt had a positive effect compared to terminator T1, and the terminator performance was significant declined, when the loop size was greater than 10 nt (Fig. 3B). Our results indicated that the loop size of intrinsic terminator stem-loop is a key factor affecting terminator performance.

### Effect of U-tract length on intrinsic terminator performance

**Fig. 4:**
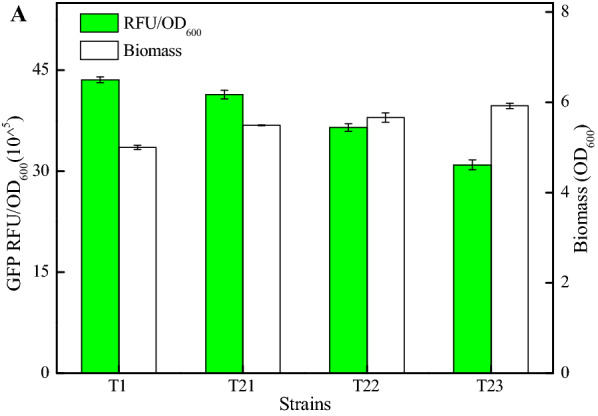
Effect of different U-tract lengths on intrinsic terminator T1. **A** Green fluorescence intensities and cell biomasses of recombinant strains with different terminators (T21–T23)

Previously, Cui et al., have reported that the length of U-tract affects termination efficiency in *B. subtilis* 168, and the best performance was attained when the length of U-tract was 6–8 nt (Cui et al. 2021). Here, different terminators (T21–T23) with reduced lengths of U-tract were designed basing on terminator T1 (Table 1), as well as the recombinant strains DW2/YG-(T21-T23). Our results found that the relative fluorescence intensities (RFU/OD_600_, GFP) mediated by T21, T22, and T23 were 41.39 × 10^^5^, 36.48 × 10^^5^, and 30.94 × 10^^5^ (Fig. 4A), decreased by 5.03%, 16.29%, and 29.00% compared to terminator T1 (43.58 × 10^^5^), respectively, indicated that reducing the length of U-tract had the negative effect on terminator strength, which was consistent with the previous results (Cui et al. 2021). Meanwhile, our results implied that U-tract tail of intrinsic terminator is essential for maintaining terminator performance in different genetic backgrounds.

Manual design and verification of terminator T24.

In prokaryotic system, although researchers identified and explored the influencing factors of intrinsic terminator, systematical optimization has not been conducted to improve terminator performance (He et al. 2020; Cui et al. 2021; Chen et al. 2013). Based on the above results, terminator T1 was further optimized by the combination strategy, considering these four factors, the distance between stop codon and terminator, GC content at the bottom of stem-loop structure, loop size and U-tract length, and the optimal terminator T24 was artificially designed (Table 1), and the T7 terminator (T7^P^), which commonly used for protein expression in *E. coli*, was acted as the reference (Mairhofer et al. 2015), attaining recombinant strains DW2/YG-T24 and DW2/YG-T7^P^, respectively.

**Fig. 5:**
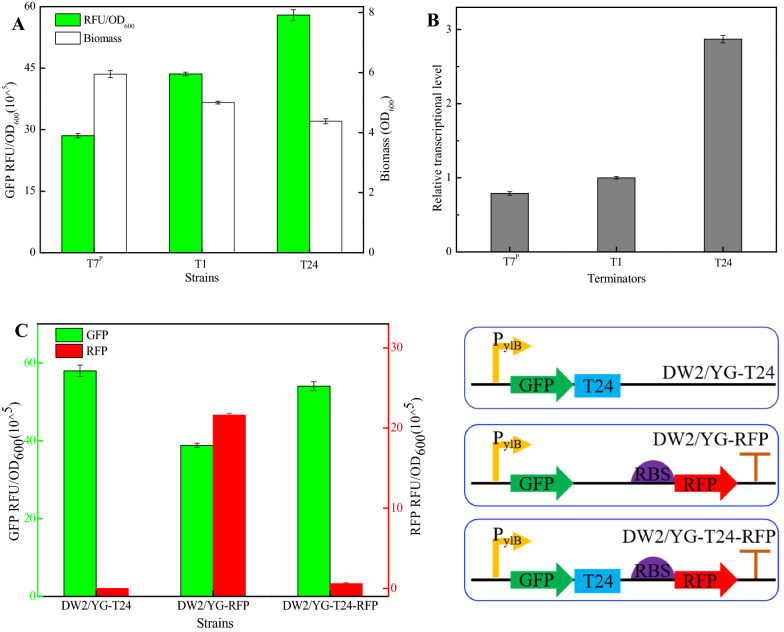
Manually design and verification of the best terminator T24. **A** Green fluorescence intensities and cell biomasses of strains with different terminators (T1, T24, and T7^P^). **B** Relative transcriptional levels of *gfp*. **C:** Determination of termination efficiency of terminator T24

Our results found that green fluorescence intensity of DW2/YG-T24 was 57.96 × 10^^5^, increased by 33.00% compared to DW2/YG-T1; however, low fluorescence intensity was attained by DW2/YG-T7^P^ (28.51 × 10^^5^) (Fig. 5A), indicating that the performance of terminator T24 is better than that of commonly used phage terminator, and the results of fluorescence intensities were consistent with those of gene transcription levels in Fig. 5B. Therefore, these results indicated that the termination strength could be effectively enhanced by portfolio-optimizing influence factors of terminator. Subsequently, the termination efficiency of T24 was determined by dual-reporter system, and the relative green and red fluorescence intensities of DW2/YG-T24-RFP were 54.02 × 10^^5^ and 0.61 × 10^^5^, respectively, and termination efficiency of T24 reached 97.97%, significant higher than that of T1 (87.81%) (Fig. 5C), which is greater than those of most terminators reported in *Bacillus spp* (Cui et al. 2021). Therefore, a better α-amylase terminator T24 was obtained by portfolio optimization, and this research provided a simple and efficient strategy for artificially designing terminators with different strengths.

### Application of terminator T24 for protein expression

**Fig. 6:**
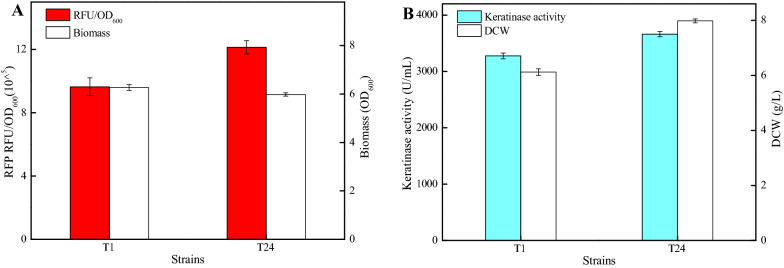
Application of terminator T24 in protein expression. **A** RFP fluorescence intensities and cell biomasses of recombinant strains with different terminators (T1, T24). **B** Keratinase activities and cell biomasses of recombinant strains with different terminators (T1, T24)

Although several researches have been focused on the identification, characterization, and artificial re-design of natural terminator in *B. subtilis*, none of them have been evaluated in protein expression (Cui et al. 2021; de Hoon et al. 2005). Here, the optimal terminator T24 was applied in protein expression, with red fluorescent protein and keratinase as target proteins. Keratinase is a family of proteases, which can specifically degrade insoluble keratin substrates, and has great application prospects in the fields of feed, leather processing, and textile. Recombinant strains DW2/P43-RFP-T24 and DW2/UTR12-KER-T24 were constructed, as well as control strains DW2/P43-RFP-T1 and DW2/UTR12-KER-T1. As shown in Fig. 6, fluorescence intensities (RFU/OD_600_, RFP) mediated by terminators T1 and T24 were 9.64 × 10^^5^ and 12.14 × 10^^5^, respectively, indicating the higher expression level of RFP produced by DW2/P43-RFP-T24 (Fig. 6A). Moreover, keratinase activity of DW2/UTR12-KER-T24 reached 3661.99 U/mL, increased by 11.78% compared to DW2/UTR12-KER-T1 (3276.08 U/mL) (Fig. 6B). Taken together, all these above results indicated that the artificially designed terminator T24 was universal in protein expression, which might also be applied for other protein expression in *Bacillus*. Meantime, the expression levels of RFP were negatively correlated with cell biomasses, and the opposite results were obtained in keratinase expression assays (Fig. 6A, B). Since RFP was intracellular expressed and keratinase was secreted to the outside of cell, and the low cell biomass of DW2/P43-RFP-T24 might be due to the cellular metabolic stress caused by intracellular RFP.

## Conclusions

We firstly identified the terminator sequence of endogenous α-amylase (AmyL) from *B. licheniformis* WX-02. Then, the terminator T1 was attained and comprehensively explored at intrinsic terminator influence factors (distance between stop codon and terminator, loop size, GC content at the bottom of stem-loop structure, U-tract length); among them, the order of influence factors on the performance of intrinsic terminators was GC content at the bottom of the stem-loop structure > loop size > distance between stop codon and terminator > U-tract length. Finally, the best terminator T24 was artificial designed by combination optimization, which has universal applicability in different genetic backgrounds. This study not only expanded the synthetic biology toolbox of *B. licheniformis* but also provided a reference for the artificial design of plug-and-play terminator library.

### Supplementary Information

**Additional file1: Table S1.** Strains and plasmids used in this study. **Table S2.** Primers used in this study. **Table S3.** The effects of GC content at the bottom of stem-loop structure, Gibbs free energy ΔG, U-tract length on terminator performances. **Table S4**. The sequences of different terminators plasmids used in this study. **Table S5**. Comparison of termination efficiency of different terminators in *Bacillus*. **Figure S1**. The secondary structure map of TamyL with a size of 501 bp. **Figure S2.** The construction map of plasmids with different terminators and dual reporter genes.

## Declarations

### Availability of data and materials

The datasets used and/or analyzed during the current study are available from the corresponding author on reasonable request.

## References

[CR1] Amarelle V, Sanches-Medeiros A, Silva-Rocha R, Guazzaroni ME (2019). Expanding the toolbox of broad host-range transcriptional terminators for *Proteobacteria* through metagenomics. ACS Synth Biol.

[CR2] Cai D, Zhu J, Li Y, Li L, Zhang M, Wang Z (2020). Systematic engineering of branch chain amino acid supply modules for the enhanced production of bacitracin from *Bacillus licheniformis*. Metab Eng Commun.

[CR3] Chen YJ, Liu P, Nielsen AA, Brophy JA, Clancy K, Peterson T (2013). Characterization of 582 natural and synthetic terminators and quantification of their design constraints. Nat Methods.

[CR4] Cui W, Lin Q, Hu R, Han L, Cheng Z, Zhang L (2021). Data-driven and in silico-assisted design of broad host-range minimal intrinsic terminators adapted for bacteria. ACS Synth Biol.

[CR5] Curran KA, Karim AS, Gupta A, Alper HS (2013). Use of expression-enhancing terminators in *Saccharomyces cerevisiae* to increase mRNA half-life and improve gene expression control for metabolic engineering applications. Metab Eng.

[CR6] de Hoon M, Makita Y, Nakai K, Miyano S (2005). Prediction of Transcriptional Terminators in *Bacillus subtilis* and Related Species. PLoS Comput Biol Prepr.

[CR7] Deng C, Lv X, Li J, Liu Y, Du G, Amaro RL (2019). Synthetic repetitive extragenic palindromic (REP) sequence as an efficient mRNA stabilizer for protein production and metabolic engineering in prokaryotic cells. Biotechnol Bioeng.

[CR8] Gibson DG, Glass JI, Lartigue C, Noskov VN, Chuang RY, Algire MA (2010). Creation of a bacterial cell controlled by a chemically synthesized genome. Science.

[CR9] He Z, Duan Y, Zhai W, Zhang X, Shi J, Zhang X (2020). Evaluating terminator strength based on differentiating effects on transcription and translation. Chem Bio Chem.

[CR10] Hironori Otakaa HI, Moritab T, Aibab H (2011). PolyU tail of rho-independent terminator of bacterial small RNAs is essential for Hfq action. Proc Natl Acad Sci USA.

[CR11] Jain S, Gupta R, Sen R (2019). Rho-dependent transcription termination in bacteria recycles RNA polymerases stalled at DNA lesions. Nat Commun.

[CR12] Jervis AJ, Carbonell P, Vinaixa M, Dunstan MS, Hollywood KA, Robinson CJ (2019). Machine learning of designed translational control allows predictive pathway optimization in *Escherichia coli*. ACS Synth Biol.

[CR13] Kang Z, Zhang M, Gao K, Zhang W, Meng W, Liu Y (2021). An L-2-hydroxyglutarate biosensor based on specific transcriptional regulator LhgR. Nat Commun.

[CR14] Knott GJ, J. A. D. (2018). CRISPR-Cas guides the future of genetic engineering. Science.

[CR15] Lesnik EA, Harold RS, Levene B, Henderson TJ, Ecker JAMADJ (2001). Prediction of rho-independent transcriptional terminators in *Escherichia coli*. Nucleic Acids Res.

[CR16] Lin MT, Wang CY, Xie HJ, Cheung CH, Hsieh CH, Juan HF (2016). Novel utilization of terminators in the design of biologically adjustable synthetic filters. ACS Synth Biol.

[CR17] Mairhofer J, Wittwer A, Cserjan-Puschmann M, Striedner G (2015). Preventing T7 RNA polymerase read-through transcription-a synthetic termination signal capable of improving bioprocess stability. ACS Synth Biol.

[CR18] Peng Z, Zhang J, Song Y, Guo R, Du G, Chen J (2021). Engineered pro-peptide enhances the catalytic activity of keratinase to improve the conversion ability of feather waste. Biotechnol Bioeng.

[CR19] Peters JM, Vangeloff AD, Landick R (2011). Bacterial transcription terminators: the RNA 3'-end chronicles. J Mol Biol.

[CR20] Pfleger BF, Pitera DJ, Smolke CD, Keasling JD (2006). Combinatorial engineering of intergenic regions in operons tunes expression of multiple genes. Nat Biotechnol.

[CR21] Ray-Soni A, Bellecourt MJ, Landick R (2016). Mechanisms of bacterial transcription termination: all good things must end. Annu Rev Biochem.

[CR22] Rossmanith J, Weskamp M, Narberhaus F (2018). Design of a temperature-responsive transcription terminator. ACS Synth Biol.

[CR23] Sauer C, Loren V, van Themaat E, Boender LGM, Groothuis D, Cruz R, Hamoen LW (2018). Exploring the nonconserved sequence space of synthetic expression modules in *Bacillus subtilis*. ACS Synth Biol.

[CR24] Sinumvayo JP, Yang S, Chen J, Du G, Kang Z (2017). Engineering and characterization of new intrinsic transcriptional terminators in *Bacillus subtilis* 168. Sheng Wu Gong Cheng Xue Bao.

[CR25] Uwimana N, Collin P, Jeronimo C, Haibe-Kains B, Robert F (2017). Bidirectional terminators in *Saccharomyces cerevisiae* prevent cryptic transcription from invading neighboring genes. Nucleic Acids Res.

[CR26] Xiao J, Peng B, Su Z, Liu A, Hu Y, Nomura CT (2020). Facilitating protein expression with portable 5'-UTR secondary structures in *Bacillus licheniformis*. ACS Synth Biol.

[CR27] Zong Y, Zhang HM, Lyu C, Ji X, Hou J, Guo X (2017). Insulated transcriptional elements enable precise design of genetic circuits. Nat Commun.

