# Supplementary material for: Minimization and optimization of α-amylase terminator for heterologous protein production in Bacillus licheniformis
Source: Bioresour Bioprocess. 2022 Oct 10;9(1):108. doi: 10.1186/s40643-022-00597-1 (PMC10992175; doi:10.1186/s40643-022-00597-1)

**Minimization and optimization of α-amylase terminator for heterologous protein production in *Bacillus licheniformis***

Yi Rao ^a^, Jingyao Yang ^a^, Jiaqi Wang ^a^, Xinyuan Yang ^a^, Mengxi Zhang ^a^, Yangyang Zhan ^a^, Xin Ma ^a^, Dongbo Cai ^a^, Zhangqian Wang ^b*^, Shouwen Chen ^a, c*^

*^a^ State Key Laboratory of Biocatalysis and Enzyme Engineering*, *Environmental Microbial Technology Center of Hubei Province, College of Life Sciences, Hubei University, Wuhan, 430062, PR China*

*^b^National R&D Center for Se-rich Agricultural Products Processing, Hubei Engineering Research Center for Deep Processing of Green Se-rich Agricultural Products, School of Modern Industry for Selenium Science and Engineering, Wuhan Polytechnic University, Wuhan 430023, PR China*

*^c^ Fujian Provincial Key Laboratory of Eco-Industrial Green Technology, College of Ecological and Resource Engineering, Wuyi University, Wuyishan 354300, PR China*

^*^Corresponding author: Prof. Shouwen Chen and Prof. Zhangqian Wang

Tel./fax.: +86 027-88666081.

E-mail address: [mel212@126.com](mailto:mel212@126.com) (S. Chen) and wzqsnu@whpu.edu.cn (Z. Wang)

Postal address: 368 Youyi Avenue, Wuchang District, Wuhan 430062, Hubei, PR China

**Table S1 Strains and plasmids** used in this study

| Strains | Characteristics | Source |
| --- | --- | --- |
| *Escherichia coli* |  |  |
| DH5α | F^–^ Φ80d/*lac*ZΔM15, Δ(*lacZYA-argF*) U169, *recA*1, *endA*1, *hsdR*17 (*r*_K_^–^, *m*_K_^+^), *phoA*, *supE*44, λ^–^, *thi*-1, *gyrA*96, *relA*1 | Stored in this lab |
| *Bacillus licheniformis* |  |  |
| DW2 | Wide-type | Stored in lab |
| DW2/300 | DW2 contains plasmid pHY300PLK | Stored in lab |
| DW2/pP_ylB_-GFP-TamyL | DW2 contains plasmid pHY-P_ylB_-GFP-TamyL | This study |
| DW2/pP_ylB_-GFP-T1 | DW2 contains plasmid pHY-P_ylB_-GFP-T1 | This study |
| DW2/pP_ylB_-GFP-T2 | DW2 contains plasmid pHY-P_ylB_-GFP-T2 | This study |
| DW2/pP_ylB_-GFP-T3 | DW2 contains plasmid pHY-P_ylB_-GFP-T3 | This study |
| DW2/pP_ylB_-GFP-T4 | DW2 contains plasmid pHY-P_ylB_-GFP-T4 | This study |
| DW2/pP_ylB_-GFP-T5 | DW2 contains plasmid pHY-P_ylB_-GFP-T5 | This study |
| DW2/pP_ylB_-GFP-T6 | DW2 contains plasmid pHY-P_ylB_-GFP-T6 | This study |
| DW2/pP_ylB_-GFP-T7 | DW2 contains plasmid pHY-P_ylB_-GFP-T7 | This study |
| DW2/pP_ylB_-GFP-T8 | DW2 contains plasmid pHY-P_ylB_-GFP-T8 | This study |
| DW2/pP_ylB_-GFP-T9 | DW2 contains plasmid pHY-P_ylB_-GFP-T9 | This study |
| DW2/pP_ylB_-GFP-T10 | DW2 contains plasmid pHY-P_ylB_-GFP-T10 | This study |
| DW2/pP_ylB_-GFP-T11 | DW2 contains plasmid pHY-P_ylB_-GFP-T11 | This study |
| DW2/pP_ylB_-GFP-T12 | DW2 contains plasmid pHY-P_ylB_-GFP-T12 | This study |
| DW2/pP_ylB_-GFP-T13 | DW2 contains plasmid pHY-P_ylB_-GFP-T13 | This study |
| DW2/pP_ylB_-GFP-T14 | DW2 contains plasmid pHY-P_ylB_-GFP-T14 | This study |
| DW2/pP_ylB_-GFP-T15 | DW2 contains plasmid pHY-P_ylB_-GFP-T15 | This study |
| DW2/pP_ylB_-GFP-T16 | DW2 contains plasmid pHY-P_ylB_-GFP-T16 | This study |
| DW2/pP_ylB_-GFP-T17 | DW2 contains plasmid pHY-P_ylB_-GFP-T17 | This study |
| DW2/pP_ylB_-GFP-T18 | DW2 contains plasmid pHY-P_ylB_-GFP-T18 | This study |
| DW2/pP_ylB_-GFP-T19 | DW2 contains plasmid pHY-P_ylB_-GFP-T19 | This study |
| DW2/pP_ylB_-GFP-T20 | DW2 contains plasmid pHY-P_ylB_-GFP-T20 | This study |
| DW2/pP_ylB_-GFP-T21 | DW2 contains plasmid pHY-P_ylB_-GFP-T21 | This study |
| DW2/pP_ylB_-GFP-T22 | DW2 contains plasmid pHY-P_ylB_-GFP-T22 | This study |
| DW2/pP_ylB_-GFP-T23 | DW2 contains plasmid pHY-P_ylB_-GFP-T23 | This study |
| DW2/pP_ylB_-GFP-T24 | DW2 contains plasmid pHY-P_ylB_-GFP-T24 | This study |
| DW2/pP_ylB_-GFP-T7^r^ | DW2 contains plasmid pHY-P_ylB_-GFP-T7^r^ | This study |
| DW2/pP_ylB_-GFP-RFP | DW2 contains plasmid pHY-P_ylB_-GFP-RFP | This study |
| DW2/pP_ylB_-GFP-T1-RFP | DW2 contains plasmid pHY-P_ylB_-GFP-T1-RFP | This study |
| DW2/pP_ylB_-GFP-T24-RFP | DW2 contains plasmid pHY-P_ylB_-GFP-T24-RFP | This study |
| DW2/pP43-RFP-T1 | DW2 contains plasmid pHY-P43-RFP-T1 | This study |
| DW2/pP43-RFP-T24 | DW2 contains plasmid pHY-P43-RFP-T24 | This study |
| DW2/pUTR12-KER-T1 | DW2 contains plasmid pHY-UTR12-KER-T1 | This study |
| DW2/pUTR12-KER-T24 | DW2 contains plasmid pHY-UTR12-KER-T24 | This study |
| Plasmids |  |  |
| pHY300PLK | *E. coli*-*Bacillus* shuttle vector; Ampr in *E. coli*,Tcr in both E. coli and *B. licheniformis* | Stored in lab |
| pHY-P_ylB_-GFP-TamyL | pHY300PLK contains the P_ylB_-GFP-TamyL expression cassette | Stored in lab |
| pHY-P_ylB_-GFP-T1 | pHY300PLK contains the P_ylB_-GFP-T1 expression cassette | This study |
| pHY-P_ylB_-GFP-T2 | pHY300PLK contains the P_ylB_-GFP-T2 expression cassette | This study |
| pHY-P_ylB_-GFP-T3 | pHY300PLK contains the P_ylB_-GFP-T3 expression cassette | This study |
| pHY-P_ylB_-GFP-T4 | pHY300PLK contains the P_ylB_-GFP-T4 expression cassette | This study |
| pHY-P_ylB_-GFP-T5 | pHY300PLK contains the P_ylB_-GFP-T5 expression cassette | This study |
| pHY-P_ylB_-GFP-T6 | pHY300PLK contains the P_ylB_-GFP-T6 expression cassette | This study |
| pHY-P_ylB_-GFP-T7 | pHY300PLK contains the P_ylB_-GFP-T7 expression cassette | This study |
| pHY-P_ylB_-GFP-T8 | pHY300PLK contains the P_ylB_-GFP-T8 expression cassette | This study |
| pHY-P_ylB_-GFP-T9 | pHY300PLK contains the P_ylB_-GFP-T9 expression cassette | This study |
| pHY-P_ylB_-GFP-T10 | pHY300PLK contains the P_ylB_-GFP-T10 expression cassette | This study |
| pHY-P_ylB_-GFP-T11 | pHY300PLK contains the P_ylB_-GFP-T11 expression cassette | This study |
| pHY-P_ylB_-GFP-T12 | pHY300PLK contains the P_ylB_-GFP-T12 expression cassette | This study |
| pHY-P_ylB_-GFP-T13 | pHY300PLK contains the P_ylB_-GFP-T13 expression cassette | This study |
| pHY-P_ylB_-GFP-T14 | pHY300PLK contains the P_ylB_-GFP-T14 expression cassette | This study |
| pHY-P_ylB_-GFP-T15 | pHY300PLK contains the P_ylB_-GFP-T15 expression cassette | Stored in lab |
| pHY-P_ylB_-GFP-T16 | pHY300PLK contains the P_ylB_-GFP-T16 expression cassette | This study |
| pHY-P_ylB_-GFP-T17 | pHY300PLK contains the P_ylB_-GFP-T17 expression cassette | This study |
| pHY-P_ylB_-GFP-T18 | pHY300PLK contains the P_ylB_-GFP-T18 expression cassette | This study |
| pHY-P_ylB_-GFP-T19 | pHY300PLK contains the P_ylB_-GFP-T19 expression cassette | This study |
| pHY-P_ylB_-GFP-T20 | pHY300PLK contains the P_ylB_-GFP-T20 expression cassette | This study |
| pHY-P_ylB_-GFP-T21 | pHY300PLK contains the P_ylB_-GFP-T21 expression cassette | This study |
| pHY-P_ylB_-GFP-T22 | pHY300PLK contains the P_ylB_-GFP-T22 expression cassette | This study |
| pHY-P_ylB_-GFP-T23 | pHY300PLK contains the P_ylB_-GFP-T23 expression cassette | This study |
| pHY-P_ylB_-GFP-T24 | pHY300PLK contains the P_ylB_-GFP-T24 expression cassette | This study |
| pHY-P_ylB_-GFP-T7^r^ | pHY300PLK contains the P_ylB_-GFP-T7^r^ expression cassette | This study |
| pHY-P_ylB_-GFP-RFP | pHY300PLK contains the P_ylB_-GFP-RBS-RFP-TamyL expression cassette | This study |
| pHY-P_ylB_-GFP-T1-RFP | pHY300PLK contains the P_ylB_-GFP-T1-RBS-RFP-TamyL expression cassette | This study |
| pHY-P_ylB_-GFP-T24-RFP | pHY300PLK contains the P_ylB_-GFP-T24-RBS-RFP-TamyL expression cassette | This study |
| pHY-P43-RFP-T1 | pHY300PLK contains the P43-RFP-T1 expression cassette | This study |
| pHY-P43-RFP-T24 | pHY300PLK contains the P43-RFP-T1 expression cassette | This study |
| pHY-PUTR12-KER-T1 | pHY300PLK contains the PUTR12-KER-T1 expression cassette | This study |
| pHY-PUTR12-KER-T24 | pHY300PLK contains the PUTR12-KER-T24 expression cassette | This study |

**Table S2 Primer**s used in this study

| Primers | Sequence of primer (5' to 3') |
| --- | --- |
| pHY-F | GTTTATTATCCATACCCTTAC |
| pHY-R | CAGATTTCGTGATGCTTGTC |
| T1-F | TAAAAAAACGGATTTCCTTCAGGAAATCCGTTACTTGTACAGCTCGTCC |
| T1-R | CGGATTTCCTGAAGGAAATCCGTTTTTTTATCTAGAAGCTTGGGCAAAGCG |
| T2-F | ATTAATTATAAAATGTAATCAAATTACTTGTACAGCTCGTCC |
| T2-R | TTTGATTACATTTTATAATTAATTCTAGAAGCTTGGGCAAAGCG |
| T3-F | AAGTCCTTCCTGAGGGCTGATGACACTTTGTTTACTTGTACAGCTCGTCC |
| T3-R | AGCCCTCAGGAAGGACTTGCTGACAGTTTGATCTAGAAGCTTGGGCAAAGCG |
| T4-F | CACAGTCTCAGGATGATTGATCACCCGCGATACCGTCTTACTTGTACAGCTCGTCC |
| T4-R | ACGGTATCGCGGGTGATCAATCATCCTGAGACTGTGTCTAGAAGCTTGGGCAAAGCG |
| T5-F | CTGATTCCCGTTAACAGATTCATTTACTTGTACAGCTCGTCC |
| T5-R | ATGAATCTGTTAACGGGAATCAGTCTAGAAGCTTGGGCAAAGCG |
| T6-F | AAAGCATAATGATGACGGTCCAGCTCGCG TTACTTGTACAGCTCGTCC |
| T6-R | ACCGTCATCATTATGCTTTGCAGCTTGTCTCTAGAAGCTTGGGCAAAGCG |
| T7-F | TTCCTTCAGGAAATCCGGTTACTTGTACAGCTCGTCC |
| T7-R | CCGGATTTCCTGAAGGAA |
| T8-F | CTTCAGGAAATCCGGGGGTTACTTGTACAGCTCGTCC |
| T8-R | CCCCCGGATTTCCTGAAGGAA |
| T9-F | TCAGGAAATCCGGGGGGGTTACTTGTACAGCTCGTCC |
| T9-R | CCCCCCCGGATTTCCTGAAGGAA |
| T10-F | CAGGAAATCCGGGGGGGGGTTACTTGTACAGCTCGTCC |
| T10-R | CCCCCCCCCGGATTTCCTGAAGGAA |
| T11-F | GAAATCCGGGGGGGGGGGTTACTTGTACAGCTCGTCC |
| T11-R | CCCCCCCCCCCGGATTTCCTGAAGGAA |
| T12-F | AATCCGGGGGGGGGGGGGTTACTTGTACAGCTCGTCC |
| T12-R | CCCCCCCCCCCCCGGATTTCCTGAAGGAA |
| T13-F | CGGGGGGGGGGGGGGGGGTTACTTGTACAGCTCGTCC |
| T13-R | CCCCCCCCCCCCCCCCCGGATTTCCTGAAGGAA |
| T14-F | GGGGGGGGGGGGGGGGGGTTACTTGTACAGCTCGTCC |
| T14-R | CCCCCCCCCCCCCCCCCCCCCGGATTTCCTGAAGGAA |
| T15-F | TAAAAAAACGGCGCATTTCCTTCAGGAAATGCGCCGTTACTTGTACAGCTCGTCC |
| T15-R | CGGCGCATTTCCTGAAGGAAATGCGCCGTTTTTTTATCTAGAAGCTTGGGCAAAGCG |
| T16-F | TAAAAAAACATATTTCCTTCAGGAAATATGTTACTTGTACAGCTCGTCC |
| T16-R | CATATTTCCTGAAGGAAATATGTTTTTTTATCTAGAAGCTTGGGCAAAGCG |
| T17-F | TAAAAAAACGGATTTCCGGTCAAGGAAATCCGTTACTTGTACAGCTCGTCC |
| T17-R | CGGATTTCCTTGACCGGAAATCCGTTTTTTTATCTAGAAGCTTGGGCAAAGCG |
| T18-F | TAAAAAAACGGATTTCCGGGTCAAAGGAAATCCGTTACTTGTACAGCTCGTCC |
| T18-R | CGGATTTCCTTTGACCCGGAAATCCGTTTTTTTATCTAGAAGCTTGGGCAAAGCG |
| T19-F | TAAAAAAACGGATTTCCGGGGTCAAAAGGAAATCCGTTACTTGTACAGCTCGTCC |
| T19-R | CGGATTTCCTTTTGACCCCGGAAATCCGTTTTTTTA TCTAGAAGCTTGGGCAAAGCG |
| T20-F | TAAAAAAACGGATTTCCGGGGGTCAAAAAGGAAATCCGTTACTTGTACAGCTCGTCC |
| T20-R | GGATTTCCTTTTTGACCCCCGGAAATCCGTTTTTTTATCTAGAAGCTTGGGCAAAGCG |
| T21-F | TAGAAAAACGGATTTCCTTCAGGAAATCCGTTACTTGTACAGCTCGTCC |
| T21-R | CGGATTTCCTGAAGGAAATCCGTTTTTCTATCTAGAAGCTTGGGCAAAGCG |
| T22-F | TAGAGAAACGGATTTCCTTCAGGAAATCCGTTACTTGTACAGCTCGTCC |
| T22-R | CGGATTTCCTGAAGGAAATCCGTTTCTCTATCTAGAAGCTTGGGCAAAGCG |
| T23-F | TAGAGAACCGGATTTCCTTCAGGAAATCCGTTACTTGTACAGCTCGTCC |
| T23-R | CGGATTTCCTGAAGGAAATCCGGTTCTCTATCTAGAAGCTTGGGCAAAGCG |
| T24-F | CATTTCCGGGTCAAAGGAAATGCGCCGGGGGGGGGGGTTACTTGTACAGCTCGTCC |
| T24-R | CATTTCCTTTGACCCGGAAATGCGCCGTTTTTTTATCTAGAAGCTTGGGCAAAGCG |
| T7^r^-F | CTCAAGACCCGTTTAGAGGCCCCAAGGGGTTTTACTTGTACAGCTCGTCC |
| T7^r^-R | TCTAAACGGGTCTTGAGGGGTTTTTTGCTTCTAGAAGCTTGGGCAAAGCG |
| T1R-F | TAAAAAAACGGATTTCCTTCAGGAAATCCGTTATTTATATAGTTCATCC |
| T1R-R | GATTTCCTGAAGGAAATCCGTTTTTTTATCTAGAAGCTTGGGCAAAGCG |
| T1K-F | TAAAAAAACGGATTTCCTTCAGGAAATCCGTTACTGAGCTGCCGCCTGTAC |
| T1K-R | GATTTCCTGAAGGAAATCCGTTTTTTTATCTAGAAGCTTGGGCAAAGCG |
| T24R-F | CATTTCCGGGTCAAAGGAAATGCGCCGGGGGGGGGGGTTATTTATATAGTTCATCC |
| T24R-R | CATTTCCTTTGACCCGGAAATGCGCCGTTTTTTTATCTAGAAGCTTGGGCAAAGCG |
| T24K-F | TTTCCGGGTCAAAGGAAATGCGCCGGGGGGGGGGGTTACTGAGCTGCCGCCTGTAC |
| T24K-R | CATTTCCTTTGACCCGGAAATGCGCCGTTTTTTTATCTAGAAGCTTGGGCAAAGCG |
| G-TF | CATGGACGAGCTGTACAAGTAA |
| T-TR | TCTAGAAGCTTGGGCAAAGCCG |
| GR-F | GACGAGCTGTACAAGTAACAACAAAGGGGGAGATTTGTATGGTTTCCAAAGGAGAAG |
| TR-R | TTTGCCCAAGCTTCTAGATTATTTATATAGTTCATCCATCC |
| T1-TF | TAAAAAAACGGATTTCCTTCAGG |
| T1R-F | AGGAAATCCGTTTTTTTACAACAAAGGGGGAGATTTGTATGGTTTCCAAAGGAGAAG |
| T24-TF | TAAAAAAACGGCGCATTTC |
| T24R-F | AAATGCGCCGTTTTTTTACAACAAAGGGGGAGATTTGTATGGTTTCCAAAGGAGAAG |
| RT-GFP-F | GGTGAACTTCAAGATCCGCC |
| RT-GFP-R | CTTGTACAGCTCGTCCATGC |
| RT-16s-F | ACCTAACCAGAAAGCCACGG |
| RT-16s-R | GTTTACGGCGTGGACTACCA |

**Table S3 The effects of GC content at the bottom of stem-loop structure, Gibbs free energy ΔG, U-tract length on terminator performances**

| Terminator | Characteristics | Strains | References |
| --- | --- | --- | --- |
| T1-T12  from  *E. coli* | The terminator sequence was dominated by GC bases, and that a high ratio of GC bases in the stem structure of terminators might be associated with a high transcription shut-down degree | *E. coli* | He et al., 2020 |
| 75 endogenous terminators | Termination efficiency of terminators with 8nt U-tract, 7 nt U-tract and 6 nt U-tract was generally higher (80%), while half of the 5 nt U-tract group had terminators with termination efficiency below 60 % | *B. subtilis* | Cui et al., 2021 |
| 582 natural and synthetic terminators | Among natural terminators or artificial terminators with different stem-loop structures, the Gibbs free energy △G of the terminator is moderately or weakly related to its termination efficiency | *E. coli* | Chen et al., 2013 |

**Table S4 The sequences of different terminators plasmids** used in this study

| Plasmids | sequences |
| --- | --- |
| pHY/PylB-GFP-Tn | TCAAGAAGATCATCTTATTAAGGGGTCTGACGCTCAGTGGAACGAAAACTCACGTTAAGGGATTTTGGTCATGAGATTATCAAAAAGGATCTTCACCTAGATCCTTTTAAATTAAAAATGAAGTTTTAAATCAATCTAAAGTATATATGAGTAAACTTGGTCTGACAGTTACCAATGCTTAATCAGTGAGGCACCTATCTCAGCGATCTGTCTATTTCGTTCATCCATAGTTGCCTGACTCCCCGTCGTGTAGATAACTACGATACGGGAGGGCTTACCATCTGGCCCCAGTGCTGCAATGATACCGCGAGACCCACGCTCACCGGCTCCAGATTTATCAGCAATAAACCAGCCAGCCGGAAGGGCCGAGCGCAGAAGTGGTCCTGCAACTTTATCCGCCTCCATCCAGTCTATTAATTGTTGCCGGGAAGCTAGAGTAAGTAGTTCGCCAGTTAATAGTTTGCGCAACGTTGTTGCCATTGCTGCAGGCATCGTGGTGTCACGCTCGTCGTTTGGTATGGCTTCATTCAGCTCCGGTTCCCAACGATCAAGGCGAGTTACATGATCCCCCATGTTGTGCAAAAAAGCGGTTAGCTCCTTCGGTCCTCCGATCGTTGTCAGAAGTAAGTTGGCCGCAGTGTTATCACTCATGGTTATGGCAGCACTGCATAATTCTCTTACTGTCATGCCATCCGTAAGATGCTTTTCTGTGACTGGTGAGTACTCAACCAAGTCATTCTGAGAATAGTGTATGCGGCGACCGAGTTGCTCTTGCCCGGCGTCAACACGGGATAATACCGCGCCACATAGCAGAACTTTAAAAGTGCTCATCATTGGAAAACGTTCTTCGGGGCGAAAACTCTCAAGGATCTTACCGCTGTTGAGATCCAGTTCGATGTAACCCACTCGTGCACCCAACTGATCTTCAGCATCTTTTACTTTCACCAGCGTTTCTGGGTGAGCAAAAACAGGAAGGCAAAATGCCGCAAAAAAGGGAATAAGGGCGACACGGAAATGTTGAATACTCATACTCTTCCTTTTTCAATATTATTGAAGCATTTATCAGGGTTATTGTCTCATGAGCGGATACATATTTGAATGTATTTAGAAAAATAAACAAATAGGGGTTCCGCGGACATTTCCCCGAAAAGTGCCACCTGACGTCTAAGAAACCATTATTATCATGACATTAACCTAGAAAGCACTAAGGAATAATTCCTTAAGGAACGTACAGACGCTTAAAAGCCTTTAAAAACGTTTTTAAGGGGTTTGTAGACAAGGTAAAGGATAAAACAGCACAATTCCAAGAAAAACACGATTTAGAACCTAAAAAGAACGAATTTGAACTAACTCATAACCGAGAGGTAAAAAAAGAACGAAGTCGAGATCAGGGAATGAGTTTATAAAATAAAAAAAGCACCTGAAAAGGTGTCTTTTTTTGATGGTTTTGAACTTGTTCTTTCTTATCTTGATACATATAGAAATAACGTCATTTTTATTTTAGTTGCTGAAAGGTGCGTTGAAGTGTTGGTATGTATGTGTTTTAAAGTATTGGAAAACCCTTAAAATTGGTTGCACAGAAAAACCCCATCTGTTAAAGTTATAAGTGACCAAACAAATAACTAAATAGATGGGGGTTTCTTTTAATATTATGTGTCCTAATAGTAGCATTTATTCAGATGAAAAATCAAGGGTTTTAGTGGACAAGACAAAAAGTGGAAAAGTGAGACCATGGAGAGAAAAGAAAATCGCTAATGTTGATTACTTTGAACTTCTGCATATTCTTGAATTTAAAAAGGCTGAAAGAGTAAAAGATTGTGCTGAAATATTAGAGTATAAACAAAATCGTGAAACAGGCGAAAGAAAGTTGTATCGAGTGTGGTTTTGTAAATCCAGGCTTTGTCCAATGTGCAACTGGAGGAGAGCAATGAAACATGGCATTCAGTCACAAAAGGTTGTTGCTGAAGTTATTAAACAAAAGCCAACAGTTCGTTGGTTGTTTCTCACATTAACAGTTAAAAATGTTTATGATGGCGAAGAATTAAATAAGAGTTTGTCAGATATGGCTCAAGGATTTCGCCGAATGATGCAATATAAAAAAATTAATAAAAATCTTGTTGGTTTTATGCGTGCAACGGAAGTGACAATAAATAATAAAGATAATTCTTATAATCAGCACATGCATGTATTGGTATGTGTGGAACCAACTTATTTTAAGAATACAGAAAACTACGTGAATCAAAAACAATGGATTCAATTTTGGAAAAAGGCAATGAAATTAGACTATGATCCAAATGTAAAAGTTCAAATGATTCGACCGAAAAATAAATATAAATCGGATATACAATCGGCAATTGACGAAACTGCAAAATATCCTGTAAAGGATACGGATTTTATGACCGATGATGAAGAAAAGAATTTGAAACGTTTGTCTGATTTGGAGGAAGGTTTACACCGTAAAAGGTTAATCTCCTATGGTGGTTTGTTAAAAGAAATACATAAAAAATTAAACCTTGATGACACAGAAGAAGGCGATTTGATTCATACAGATGATGACGAAAAAGCCGATGAAGATGGATTTTCTATTATTGCAATGTGGAATTGGGAACGGAAAAATTATTTTATTAAAGAGTAGTTCAACAAACGGGCCATATTGTTGTATAAGTGATGAAATACTGAATTTAAAACTTAGTTTATATGTGGTAAAATGTTTTAATCAAGTTTAGGAGGAATTAATTATGAAGTGTAATGAATAATGAATGTAACAGGGTTCAATTAAAAGAGGGAAGCGTATCATTAACCCTATAAACTACGTCTGCCCTCATTATTGGAGGGTGAAATGTGAATACATCCTATTCACAATCGAATTTACGACACAACCAAATTTTAATTTGGCTTTGCATTTTATCTTTTTTTAGCGTATTAAATGAAATGGTTTTGAACGTCTCATTACCTGATATTGCAAATGATTTTAATAAACCACCTGCGAGTACAAACTGGGTGAACACAGCCTTTATGTTAACCTTTTCCATTGGAACAGCTGTATATGGAAAGCTATCTGATCAATTAGGCATCAAAAGGTTACTCCTATTTGGAATTATAATAAATTGTTTCGGGTCGGTAATTGGGTTTGTTGGCCATTCTTTCTTTTCCTTACTTATTATGGCTCGTTTTATTCAAGGGGCTGGTGCAGCTGCATTTCCAGCACTCGTAATGGTTGTAGTTGCGCGCTATATTCCAAAGGAAAATAGGGGTAAAGCATTTGGTCTTATTGGATCGATAGTAGCCATGGGAGAAGGAGTCGGTCCAGCGATTGGTGGAATGATAGCCCATTATATTCATTGGTCCTATCTTCTACTCATTCCTATGATAACAATTATCACTGTTCCGTTTCTTATGAAATTATTAAAGAAAGAAGTAAGGATAAAAGGTCATTTTGATATCAAAGGAATTATACTAATGTCTGTAGGCATTGTATTTTTTATGTTGTTTACAACATCATATAGCATTTCTTTTCTTATCGTTAGCGTGCTGTCATTCCTGATATTTGTAAAACATATCAGGAAAGTAACAGATCCTTTTGTTGATCCCGGATTAGGGAAAAATATACCTTTTATGATTGGAGTTCTTTGTGGGGGAATTATATTTGGAACAGTAGCAGGGTTTGTCTCTATGGTTCCTTATATGATGAAAGATGTTCACCAGCTAAGTACTGCCGAAATCGGAAGTGTAATTATTTTCCCTGGAACAATGAGTGTCATTATTTTCGGCTACATTGGTGGGATACTTGTTGATAGAAGAGGTCCTTTATACGTGTTAAACATCGGAGTTACATTTCTTTCTGTTAGCTTTTTAACTGCTTCCTTTCTTTTAGAAACAACATCATGGTTCATGACAATTATAATCGTATTTGTTTTAGGTGGGCTTTCGTTCACCAAAACAGTTATATCAACAATTGTTTCAAGTAGCTTGAAACAGCAGGAAGCTGGTGCTGGAATGAGTTTGCTTAACTTTACCAGCTTTTTATCAGAGGGAACAGGTATTGCAATTGTAGGTGGTTTATTATCCATACCCTTACTTGATCAAAGGTTGTTACCTATGGAAGTTGATCAGTCAACTTATCTGTATAGTAATTTGTTATTACTTTTTTCAGGAATCATTGTCATTAGTTGGCTGGTTACCTTGAATGTATATAAACATTCTCAAAGGGATTTCTAAATCGTTAAGGGATCAACTTTGGGAGAGAGTTCAAAATTGATCCTTTTTTTATAACAGGAATTCCATCGTCGAACGCGCTCCATTTTATCGCACCTAGGCCTGTATGATCAACAGCTGTATCCGATTTTCTTACAGCTCCGTGCTCAACGATTCCAACATAAATGGAATGGCCAACGTGCCAGTCTTTTAGGTCGTGGATATGCCCCATGCCGTTTTGCAAAAATAATATATTCGTCTTCCCGATTCGTTCAAGCGACGAAAAAACAGATTGAAGCTGATGCTGCTTCACTGTCACGACAAGCAGGTCAAAGTCCGAATTGATACTCGTGTCCGCACTGCAATCAGCCCTGAATTCCTCCCCGCCTTTATAAAGCCGGATTCCTTCAGACTGAATGGCCGCAGCCTGTTCTTGCCGCCTCGTCACAACAGTCACGTCGTGATAAAGTGACAAATAATAGGCGCATAAAAGACCAACGGAGCCTCCGCCGATAATTCCAATTTTCATGATGTCACACCCAATTTAGCATTTACGTATTATCATAGCAGAAGTAAGAAGAAATTACTTCTCAAAGATCCCATGTGCTTAAAATTAAAGTTTAAATATTTGGATTTTTTAAATAAAGCGTTTACAATATATGTAGAAACAACAAAGGGGGAGATTTGTATGGTGAGCAAGGGCGAGGAGCTGTTCACCGGGGTGGTGCCCATCCTGGTCGAGCTGGACGGTGACGTAAACGGCCACAAGTTCAGCGTGTCCGGCGAGGGCGAGGGCGATGCCACCTACGGCAAGCTGACCCTGAAGTTCATCTGCACCACCGGCAAGCTGCCCGTGCCCTGGCCCACCCTCGTGACCACCCTGACCTACGGCGTGCAGTGCTTCAGCCGCTACCCCGACCACATGAAGCAGCACGACTTCTTCAAGTCCGCCATGCCCGAAGGCTACGTCCAGGAGCGCACCATCTTCTTCAAGGACGACGGCAACTACAAGACCCGCGCCGAGGTGAAGTTCGAGGGCGACACCCTGGTGAACCGCATCGAGCTGAAGGGCATCGACTTCAAGGAGGACGGCAACATCCTGGGGCACAAGCTGGAGTACAACTACAACAGCCACAACGTCTATATCATGGCCGACAAGCAGAAGAACGGCATCAAGGTGAACTTCAAGATCCGCCACAACATCGAGGACGGCAGCGTGCAGCTCGCCGACCACTACCAGCAGAACACCCCCATCGGCGACGGCCCCGTGCTGCTGCCCGACAACCACTACCTGAGCACCCAGTCCGCCCTGAGCAAAGACCCCAACGAGAAGCGCGATCACATGGTCCTGCTGGAGTTCGTGACCGCCGCCGGGATCACTCTCGGCATGGACGAGCTGTACAAGTAANNN...NNNGGGCAAAGCGTTTTTCCATAGGCTCCGCCCCCTGACAAGCATCACGAAATCTGACGCTCAAATCAGTGGTGGCGAAACCCGACAGGACTATAAAGATACCAGGCGTTTCCCCCTGGCGGCTCCCTCGTGCGCTCTCCTGTTCCTGCCTTTCGGTTTACCGGTGTCATTCCGCTGTTATGGCCGCGTTTGTCTCATTCCACGCCTGACACTCAGTTCCGGGTAGGCAGTTCGCTCCAAGCTGGACTGTATGCACGAACCCCCCGTTCAGTCCGACCGCTGCGCCTTATCCGGTAACTATCGTCTTGAGTCCAACCCGGAAAGACATGCAAAAGCACCACTGGCAGCAGCCACTGGTAATTGATTTAGAGGAGTTAGTCTTGAAGTCATGCGCCGGTTAAGGCTAAACTGAAAGGACAAGTTTTGGTGACTGCGCTCCTGCAAGCCAGTTACCTCGGTTCAAAGAGTTGGTAGCTCAGAGAACCTTCGAAAAACCTCCCTGCAAGGCGGTTTTTTCGTTTTCAGAGCAAGAGATTACGCGCAGACCAAAACGATC |

Note: NNN...NNN refers to different terminators sequences

**Table S5 Comparison of termination efficiency of different terminators in *Bacillus***

| Terminators | Termination efficiency (%) | Terminator types | Terminator size (bp) |
| --- | --- | --- | --- |
| *yfnB* | 93.15 | Intrinsic terminator | 50 |
| *araE* | 92.30 | Intrinsic terminator | 44 |
| *yfmR* | 90.90 | Intrinsic terminator | 42 |
| *sigA* | 88.09 | Intrinsic terminator | 47 |
| *sinR* | 87.70 | Intrinsic terminator | 55 |
| *lysS* | 85.09 | Intrinsic terminator | 54 |
| *ywlFG* | 83.67 | Intrinsic terminator | 36 |
| *yqjL* | 82.57 | Intrinsic terminator | 43 |
| *yflT* | 82.00 | Intrinsic terminator | 34 |
| *yfjN* | 80.75 | Intrinsic terminator | 46 |
| *clpP* | 78.23 | Intrinsic terminator | 46 |
| *yfmL* | 70.54 | Intrinsic terminator | 33 |
| *yfiQ* | 66.82 | Intrinsic terminator | 43 |
| *sucD* | 65.48 | Intrinsic terminator | 47 |
| *katX* | 58.07 | Intrinsic terminator | 42 |
| *yqiG* | 54.64 | Intrinsic terminator | 47 |
| **T1** | **87.87** | **Intrinsic terminator** | **30** |

**Figure S1 The secondary structure map of TamyL with a size of 501 bp**


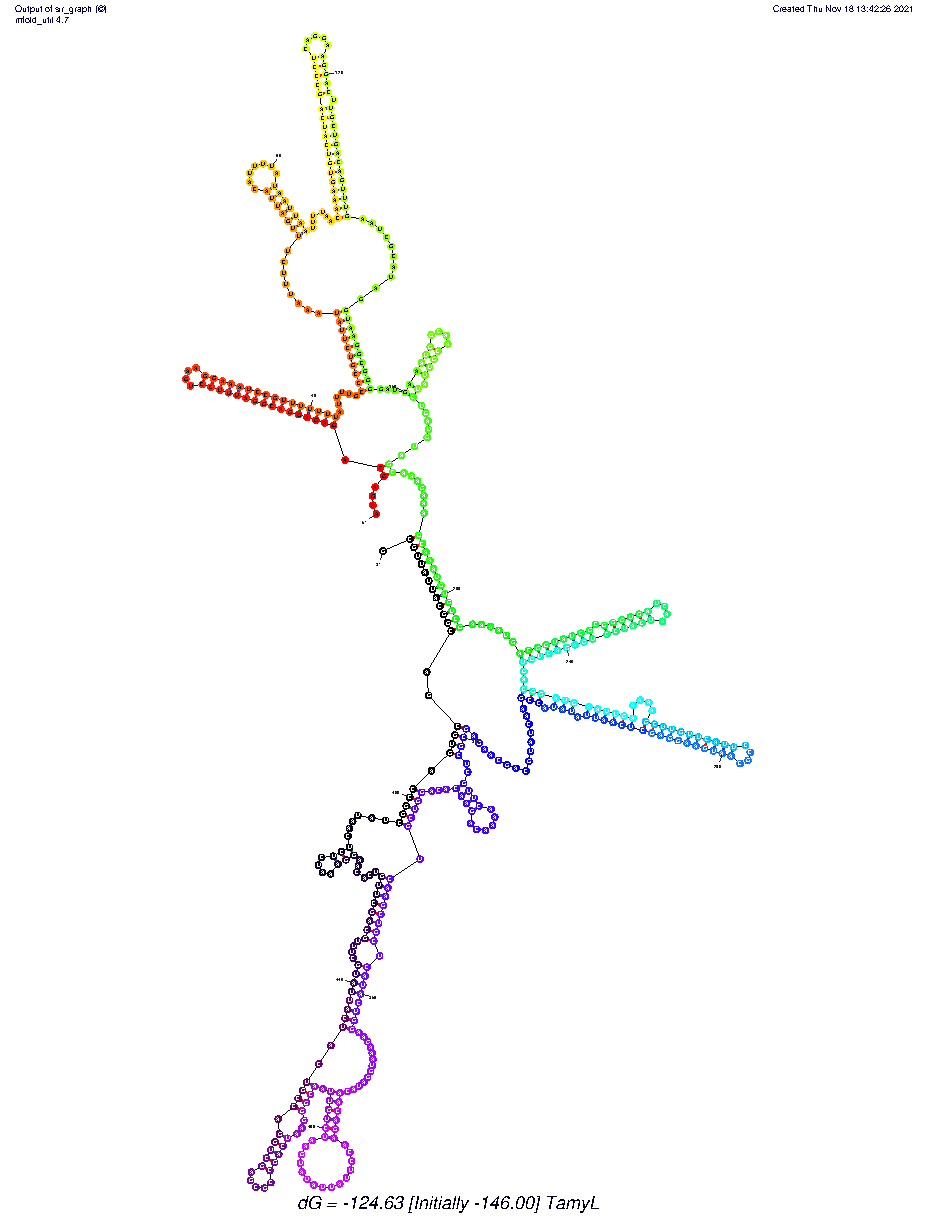


**Figure S2 The Construction map of plasmids with different terminators and dual reporter genes**


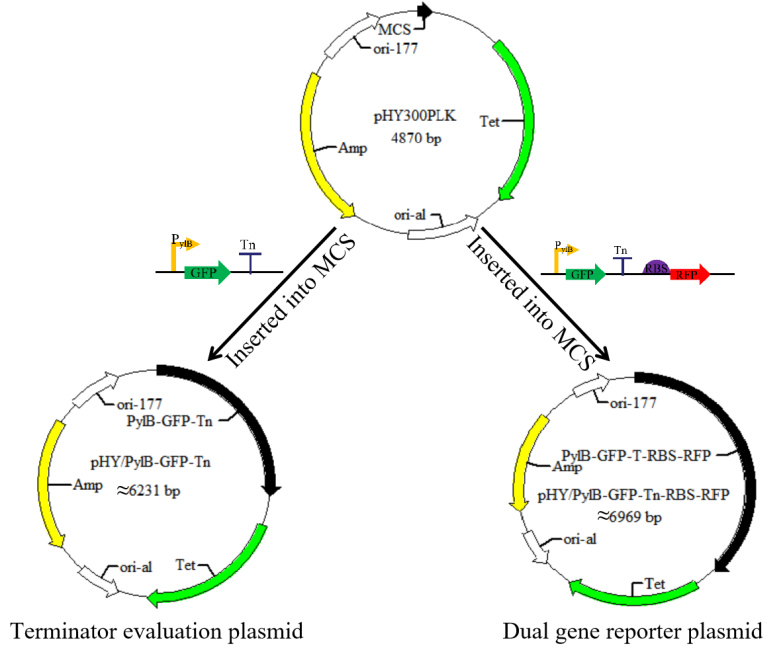

Supplement: Supplementary file 1 — Additional file1: Table S1. Strains and plasmids used in this study. Table S2. Primers used in this study. Table S3. The effects of GC content at the bottom of stem-loop structure, Gibbs free energy ΔG, U-tract length on terminator performances. Table S4. The sequences of different terminators plasmids used in this study. Table S5. Comparison of termination efficiency of different terminators in Bacillus. Figure S1. The secondary structure map of TamyL with a size of 501 bp. Figure S2. The construction map of plasmids with different terminators and dual reporter genes. [file 40643_2022_597_MOESM1_ESM.doc]
